# Carbonization of cellulose cell wall evaluated with ultraviolet microscopy†

**DOI:** 10.1039/c9ra09435k

**Published:** 2020-02-19

**Authors:** Takashi Nomura, Eiji Minami, Haruo Kawamoto

**Affiliations:** Graduate School of Energy Science, Kyoto University Yoshida-honmachi, Sakyo-ku Kyoto 606-8501 Japan kawamoto@energy.kyoto-u.ac.jp

## Abstract

This is the first study of cellulose carbonization in the interior of cell walls. Cotton cellulose was pyrolyzed under nitrogen or in aromatic solvents (benzophenone, diphenyl sulfide, and 1,3-diphenoxybenzene) at 280 °C, and cross sections of the cell walls were examined using ultraviolet (UV) microscopy. After pyrolysis under nitrogen, UV absorption caused by carbonization appeared inside the cell walls. The absorptivity of the cell interiors was homogeneous and slightly lower than that of the cell surfaces. The UV spectra had maximal absorption at *ca.* 250 nm. The spectra of model compounds and Py-GC/MS analysis data suggested that furanic and polycyclic aromatic structures were present in the carbonized products. The use of aromatic solvents decreased the yields of solid carbonized products and the UV absorptivity, which remained homogeneous throughout the cross sections. The mechanism of cellulose carbonization in cell walls is discussed along with the influence of aromatic solvents.

## Abstract 2

This is the first study of cellulose carbonization in the interior of cell walls.

## Graphical abstract

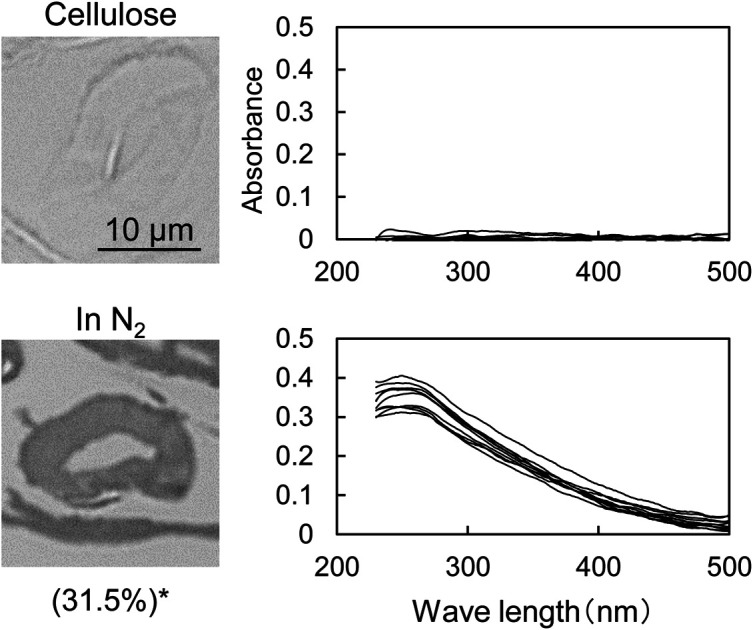

## 1. Introduction

Cellulose is the major component of the cell walls of plants. Thermochemical conversion technologies such as pyrolysis are promising ways to convert cellulose into renewable fuels, chemicals, and materials. Carbonization is a pyrolysis process that provides charcoal efficiently. Promoting carbonization produces more solid products as charcoal, while suppressing the reactions that produce more liquid or gaseous products such as bio-oil or biogas. Understanding the mechanism of carbonization allows for better control of pyrolysis reactions for practical application.

The carbonization mechanism of cellulose has been studied using various methods including infrared (IR) spectroscopy,^1–4^ changes in elemental composition,^1^ solid ^13^C-NMR,^2,4–6^ and pyrolysis-gas chromatography-mass spectrometry (Py-GC/MS).^4^ In IR analysis, characteristic signals appear at 1600 and 1700 cm^−1^ as the cellulose carbonization proceeds. These signals are assigned to conjugated unsaturated C=C bonds such as benzene rings and carbonyl groups (C=O), respectively, and are associated with dehydration reactions.^1–4^

Dehydration is the main reaction in cellulose carbonization, especially at relatively low pyrolysis temperatures (<280 °C).^7–10^ Based on the van Kreveken diagram of the elemental composition of cellulose char, Tang and Bacon^7^ determined that dehydration is the main reaction in the pyrolysis of cellulose at 200–280 °C. Scheirs *et al.*^8^ showed that dehydration of Whatman cellulose occurred from 220 °C to 350 °C, and that the total amount of water remaining was 14.3% (w/w).^8^

The solid carbonized product, the final product of dehydration, has been reported to contain benzene rings.^2,4–6,11,12^ Smith and Howard^11^ reported that benzenecarboxylic acids were obtained from cellulose char by alkaline permanganate oxidation and that the benzene ring structures were formed at temperatures above 200 °C by slow pyrolysis. Under fast pyrolysis conditions, Shafizadeh and Sekiguchi^12^ reported that benzene ring structures developed at 350–400 °C to form stable char based on the results of permanganate oxidation and solid ^13^C-NMR analysis.

Pastorova *et al.*^4^ analyzed cellulose chars prepared at different pyrolysis temperatures using Py-GC/MS. Based on the fragment structure, they reported that as carbonization progressed, the chemical structure of char changed from carbohydrate → furan → benzene. Furanoic compounds such as furfural and 5-hydroxymethylfurfural (5-HMF) have subsequently been proposed as important intermediates in the hydrothermal carbonization of reducing sugars.^13–15^ In our previous paper, a negative relationship was observed between the yield of 5-HMF and solid carbonized product (cellulose char hydrolysis residue) in the pyrolysis of cellulose in nitrogen and various aromatic solvents, which can stabilize thermal degradation products (intermediates) by forming OH–π hydrogen bonds and breaking intermolecular hydrogen bonding.^16^ These results indicate that 5-HMF is an important intermediate in cellulose carbonization under pyrolysis conditions.

These carbonization reactions proceed in cell walls composed of innumerable cellulose crystallites. For example, a single cotton fiber cell with a cross-sectional width of approximately 10–20 μm^17–19^ contains a very large number of cellulose crystallites (cross-section: 6 nm × 6 nm).^20^ Cellulose is consequently a heterogeneous material on the nanoscale. It has been reported that in pyrolysis, the molecules inside the crystallites are stable and the pyrolysis proceeds from the surface of crystallites.^21–23^ However, there is no information available on whether carbonization in the cellulose cell wall (several μm in width of cross-section) proceeds uniformly or non-uniformly.

For this purpose, there is a way to use a UV microscope with a resolution of several hundred nm. Cellulose char is frequently analyzed using optical microscopy^24^ and scanning electron microscopy (SEM),^25,26^ but the resolution of the optical microscope is not sufficient to observe the inside of the cell wall due to the intrinsic low resolution. Although the resolution of SEM is high, only the shape of the char is observed, and no inference can be made about how the carbonization progresses. UV is not the best way to analyze the chemical structure of char because of the poor resolution and less information from the UV absorptivity. By using UV microscopy, however, we can investigate the homogeneity of cellulose cell wall pyrolysis, which was validated in this article.

Using the UV microscope with the resolution of several hundred nm, the distribution of lignin within the walls of wood cells has been clearly evaluated.^27–30^ In this work, we used UV microscopy to study pyrolyzed cotton cellulose fibers. Cotton cellulose is essentially free of UV-absorbing functional groups, whereas the carbonized products with benzene and furan structures absorb UV rays. The distributions of these products can therefore be observed in cross-sections of cell walls using UV microscopy. The mechanism by which aromatic solvents suppress carbonization is also discussed by using three aromatic solvents reported in our previous study,^16^ focusing on the action inside the cell wall.

## 2. Experimental

### 2.1 Materials

Whatman No. 42 filter paper (Whatman PLC, UK, cotton) was used as a cellulose sample. Diphenyl sulfide [DPS, melting point (mp) −40 °C, boiling point (bp) 296 °C], 1,3-diphenoxybenzene (DPB, mp 166–171 °C, bp 375 °C), and benzophenone (BPH, mp 47–51 °C, bp 305 °C) were purchased from Tokyo Kasei Kogyo Co., Ltd. (Tokyo, Japan), Wako Pure Chemical Industries, Ltd. (Osaka, Japan), and Nacalai Tesque, Inc. (Kyoto, Japan), respectively, and used without purification. DPS is a thioether derivative. BPH has a carbonyl substituent that increases its reactivity with the hydrophilic surface of cellulose crystallites. Although DPB and BPH are solids at room temperature, they melted during the heating process. These solvents were stable under the pyrolysis conditions used in this study.

The UV spectra of furans and benzenes were measured using a UV-1800 spectrophotometer (Shimadzu, Japan). Furan (Nacalai Tesque), 5-HMF (Tokyo Kasei Kogyo Co.), and dibenzofuran (Tokyo Kasei Kogyo Co.) were used as model furans. Benzene (Nacalai Tesque), naphthalene (Nacalai Tesque), and anthracene (Nacalai Tesque) were used as model aromatic compounds.

### 2.2 Pyrolysis

Cellulose (38 mg dry basis) was placed in a Pyrex glass tube reactor (internal diameter 8.0 mm, wall thickness 1.0 mm, length 300 mm) that was connected to a nitrogen bag through a three-way tap. After the air inside the reactor was replaced with nitrogen (99.99%) using an aspirator, the reactor was inserted into a muffle furnace preheated to 280 °C. After heating for 58 min, the reactor was removed from the furnace and immediately cooled by a flow of air. The residue was first washed with chloroform (5 mL, five times) and subsequently with methanol (5 mL, five times), and then dried in an oven at 105 °C for 24 h.

Pyrolysis experiments in aromatic solvents were conducted in a similar manner. Cellulose was heated with aromatic solvent (400 mg) for 60 min because the presence of the aromatic solvent necessitated an additional 2 minutes of heating. This was determined from direct temperature measurements made with a fine thermocouple (0.25 mm in diameter, type K, Shin-Etsu Co., Ltd., Ibaraki, Japan).

Chemical analyses of the solutes in each solvent and the residues have been described in our previous paper.^16^

### 2.3 Microscope observations

Cellulose carbonization in cotton cell walls was observed using UV microscopy. Each residue was embedded in epoxy resin, and the samples were cut into 0.5 μm thick sections using a diamond knife mounted on a Leica Reichert Supernova Microtome (Buffalo Grove, IL, USA). The sections were placed on quartz slides, mounted with glycerin, and covered with a quartz coverslip before examination with an MSP-800 system (Carl Zeiss, Oberkochen, Germany) with a specified filter at 280 nm ± 5 nm. The morphological regions of each fraction were analyzed on a UV microspectrophotometer using photometric point-by-point measurements (spot size: 1 × 1 μm).

### 2.4 Py-GC/MS observations of the hydrolyzed residue of pyrolyzed cellulose

Cellulose pyrolyzed under nitrogen was hydrolyzed by acid hydrolysis. Aqueous H_2_SO_4_ (72 wt%, 0.1 mL) was added to the residue and the mixture was then heated at 30 °C for 60 min with frequent agitation with a glass rod. The mixture was diluted with water (2.8 mL) and then heated in an autoclave at 121 °C for 60 min. After washing with distilled water and drying, the hydrolyzed residue (solid hydrolyzed products) was analyzed using a portable Curie-point injector (JCI-22, Japan Analytical Industry, Tokyo, Japan) coupled to a Shimadzu-2010 Plus gas chromatograph (Shimadzu Corporation, Kyoto, Japan) and a Shimadzu QP 2010 Ultra mass spectrometer (Shimadzu Corporation, Kyoto, Japan). The hydrolyzed residue was pyrolyzed at 723 °C for 5 s. The instrument conditions were as follows: Agilent CPSil 8CB column (length: 30 m, diameter: 0.25 mm); 250 °C injector temperature; 1 : 50 split ratio; helium carrier gas (1.0 mL min^−1^). The column temperature was initially set at 50 °C for 3 min, after which it was ramped at 6 °C min^−1^ to 200 °C and 30 °C min^−1^ to 300 °C, before being held constant at 300 °C for 5 min. The MS scan parameters included a scan range of 35–600 *m*/*z* and a scan interval of 0.3 s.

## 3. Results and discussion

### 3.1 Cellulose carbonization in cell wall under nitrogen

**Fig. 1:**
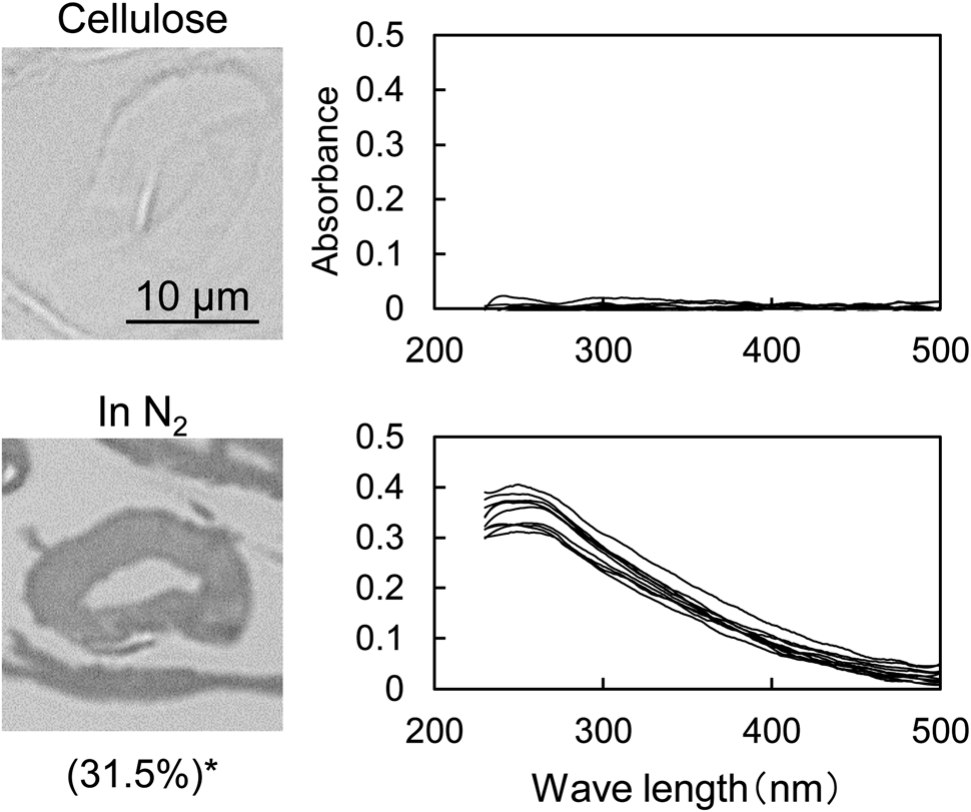
Change in the UV photomicrograph (280 nm) and UV absorption spectrum of the cross sections of cotton cellulose fiber after pyrolysis in nitrogen at 280 °C for 58 min. *Recovery rate of cellulose (cellulose base). The UV spectra were measured at ten spots selected from the cross section in the picture in a random manner excluding the edge.

Fig. 1 shows the UV photomicrographs and the absorption spectra of the cross sections of the cotton-cellulose fibers before and after pyrolysis under nitrogen. The original cellulose has no UV absorption, while the cellulose after pyrolysis absorbs UV light, and the cross section is clearly observed in the photomicrograph. The furan and benzene structures formed in the cell wall absorb UV light as discussed below. The UV absorption of the cellulose char was homogeneous within the cell wall, indicating that the carbonization reaction of cellulose occurred homogeneously within the cell wall.

**Fig. 2:**
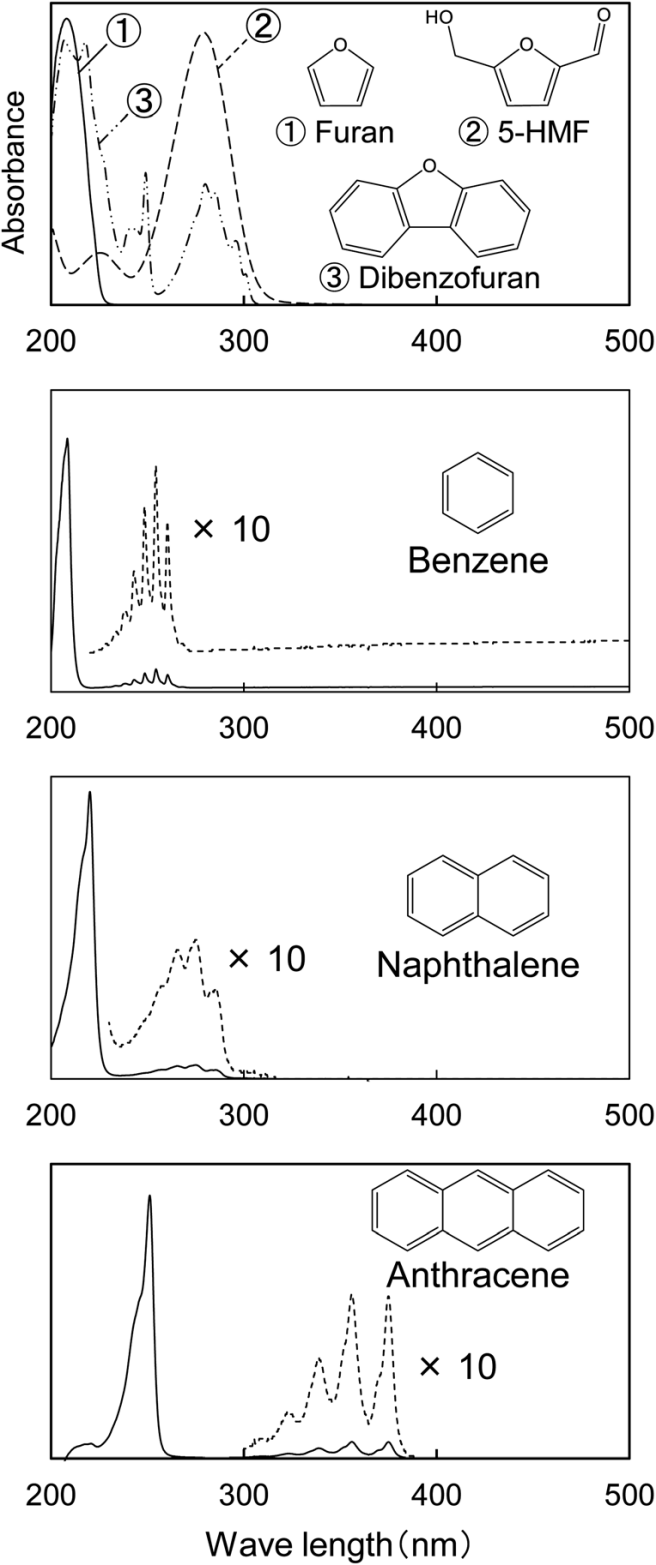
UV absorption spectra of model compounds furan, 5-HMF, dibenzofuran, benzene, naphthalene, and anthracene shown for comparison with the spectra of cellulose char.

The UV spectrum of the cellulose char (Fig. 1) has a peak at 250 nm and the absorbance gradually decreases with increasing wavelength up to around 500 nm. The UV spectra of the model compounds (*i.e.*, furan, 5-HMF, and dibenzofuran (model furans) and benzene, naphthalene and anthracene (model aromatics)) are shown in Fig. 2 for comparison. These model compounds have UV absorption peaks at around 200–300 nm, similar to cellulose char. As the conjugated system becomes larger (benzene → naphthalene → anthracene), the peak wavelength tends to shift towards the long-wavelength region.^31^ Cellulose char absorbs UV up to 500 nm, indicating that polycyclic benzene- and furan-rings are present in the cellulose char.

**Fig. 3:**
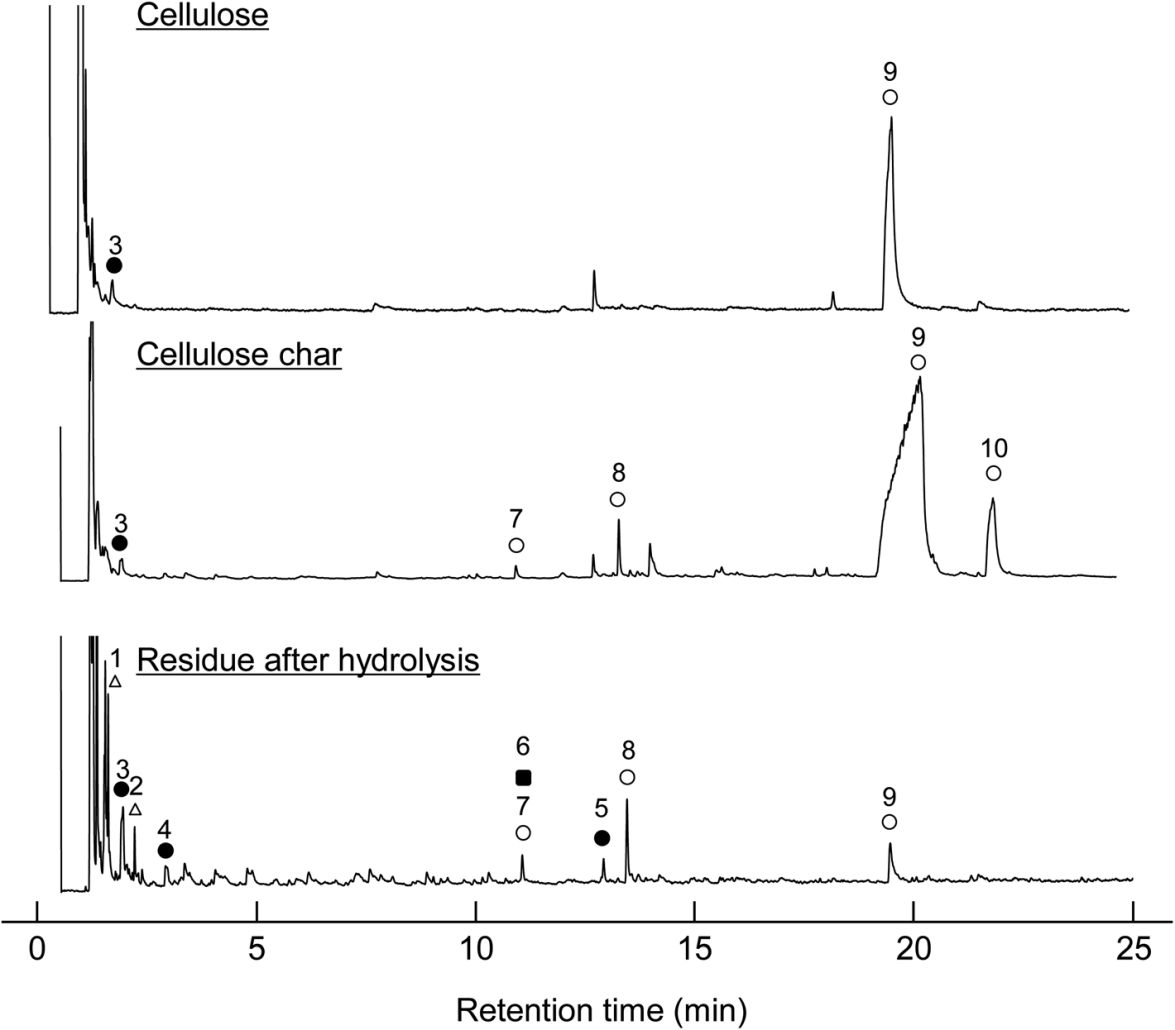
Pyrograms obtained by Py-GC/MS (764 °C/5 s) analysis of cellulose, cellulose char, and its hydrolysis residue.

**Fig. 4:**
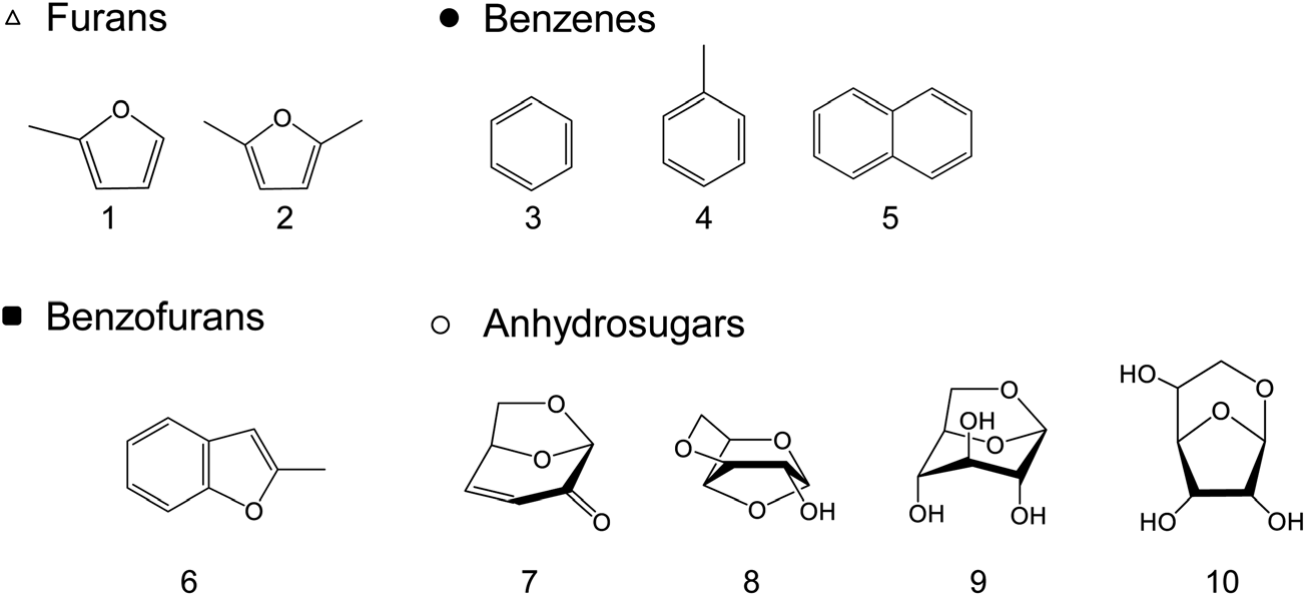
Chemical structures of the products identified by Py-GC/MS (764 °C/5 s) analysis of cellulose, cellulose char, and its hydrolysis residue. (BPH, DPS, and DPB) at 280 °C for 60 min.

When the cellulose char was hydrolyzed, the remaining cellulose was removed, and a dark brown hydrolysis residue was obtained. Fig. 3 shows the pyrograms obtained by Py-GC/MS (764 °C/5 s) analysis of cellulose, cellulose char, and the hydrolysis residue. The chemical structure assigned to each peak in the mass spectrum is shown in Fig. 4. Anhydrosugars such as levoglucosan are the major products from cellulose and cellulose char. These products may be formed directly by cellulose pyrolysis. Furans, benzenes, and benzofurans were clearly observed in the pyrogram of the hydrolysis residue. Pastorova *et al.* also reported that cellulose char obtained by pyrolysis at 270 °C and 290 °C contained furans and benzenes detected by Py-GC/MS analysis.^4^ In their study, however, large amounts of cellulose remained unreacted and it is unclear whether the furans and benzenes occurred in the solid carbonized products or in unreacted cellulose in the char. In the present study, most of the cellulose (content: 49% in cellulose char) was removed by hydrolysis before Py-GC/MS analysis. Consequently, our results indicate that the solid carbonized products in cellulose char prepared at 280 °C contained furan and benzene rings that absorb UV light.

### 3.2 Influence of aromatic solvent

**Fig. 5:**
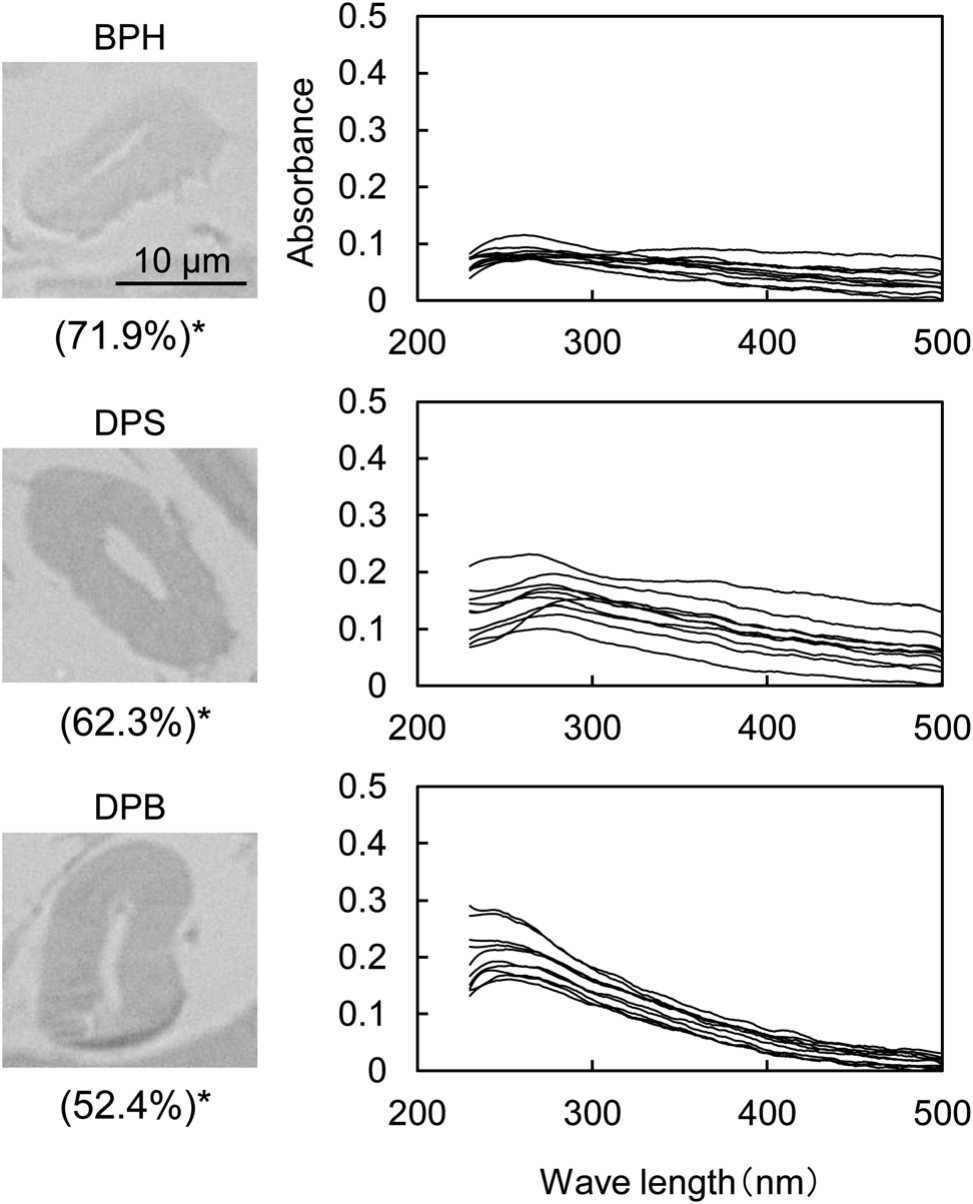
UV photomicrographs (280 nm) and UV absorption spectra of the cross sections of cotton cellulose fiber pyrolyzed in aromatic solvents (BPH, DPS and DPB) at 280 °C for 60 min. *Recovery rate of cellulose (cellulose base). The UV spectra were measured at ten spots selected from the cross section in the picture in a random manner excluding the edge.

**Fig. 6:**
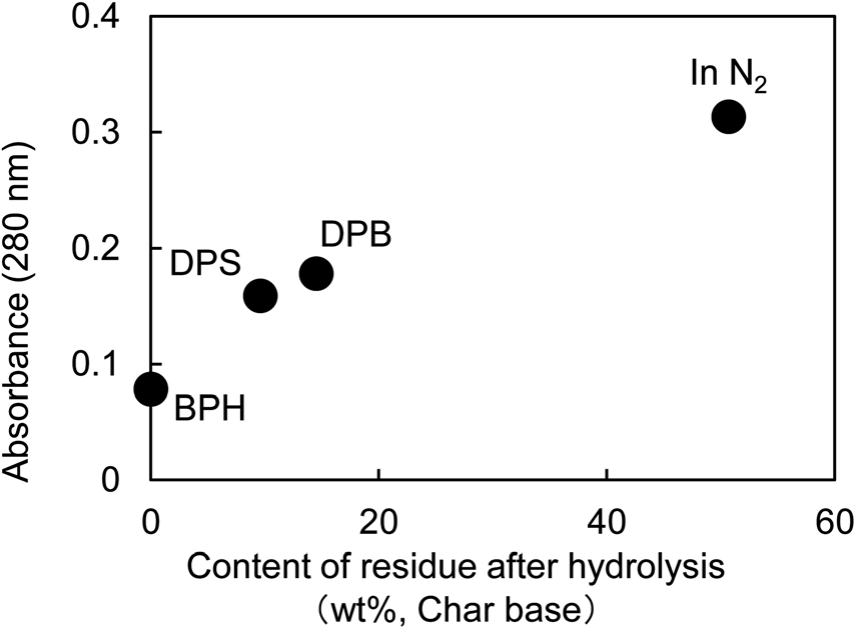
Correlation between UV absorbance at 280 nm and yield of char hydrolysis residue.

Fig. 5 shows the UV micrographs and spectra of the cross sections of cellulose fiber pyrolyzed in aromatic solvents. The UV absorptivity of cellulose pyrolyzed in aromatic solvent is lower than that of cellulose pyrolyzed under nitrogen, and decreases in the order N_2_ > DPB > DPS > BPH, which is the same order as the yield of the hydrolysis residue of char (Fig. 6). As the yield of hydrolysis residue (%, char basis) decreases, so does the UV absorbance at 280 nm. These results confirm that the UV absorption is caused by the solid carbonized products formed in cellulose char.

The UV absorptivity was homogeneous within the cross section of the cellulose fiber after pyrolysis in aromatic solvents, suggesting that the aromatic solvents penetrated into the cell walls and effectively inhibited the carbonization reaction. Because the shapes of the UV spectra (Fig. 5), which have absorption maxima at 250 nm, are very similar to those of cellulose pyrolyzed under nitrogen, similar carbonization reactions must take place under nitrogen and in the aromatic solvent, though the frequency of these reactions is lower in the aromatic solvent. In our previous study we also found that the IR spectra of hydrolysis residue obtained under nitrogen and in aromatic solvent were similar.^16^

Aromatic solvents stabilize levoglucosan, the primary cellulose pyrolysis product, against secondary pyrolysis reactions.^32,33^ Hydrogen bonding between levoglucosan molecules promotes secondary reactions by causing them to act as acid and base catalysts.^33–36^ The formation of OH/π-hydrogen bonding between levoglucosan OHs and the π electrons of benzene rings breaks the hydrogen bonding between levoglucosan molecules and so stabilizes levoglucosan.^33^ The yields of levoglucosan and 5-HMF increased significantly with the inhibition of solid carbonized product formation during the pyrolysis of cellulose in aromatic solvents, suggesting that these compounds are intermediates in the formation of solid carbonized products.^16^

### 3.3 Carbonization mechanism of cellulose fiber

**Fig. 7:**
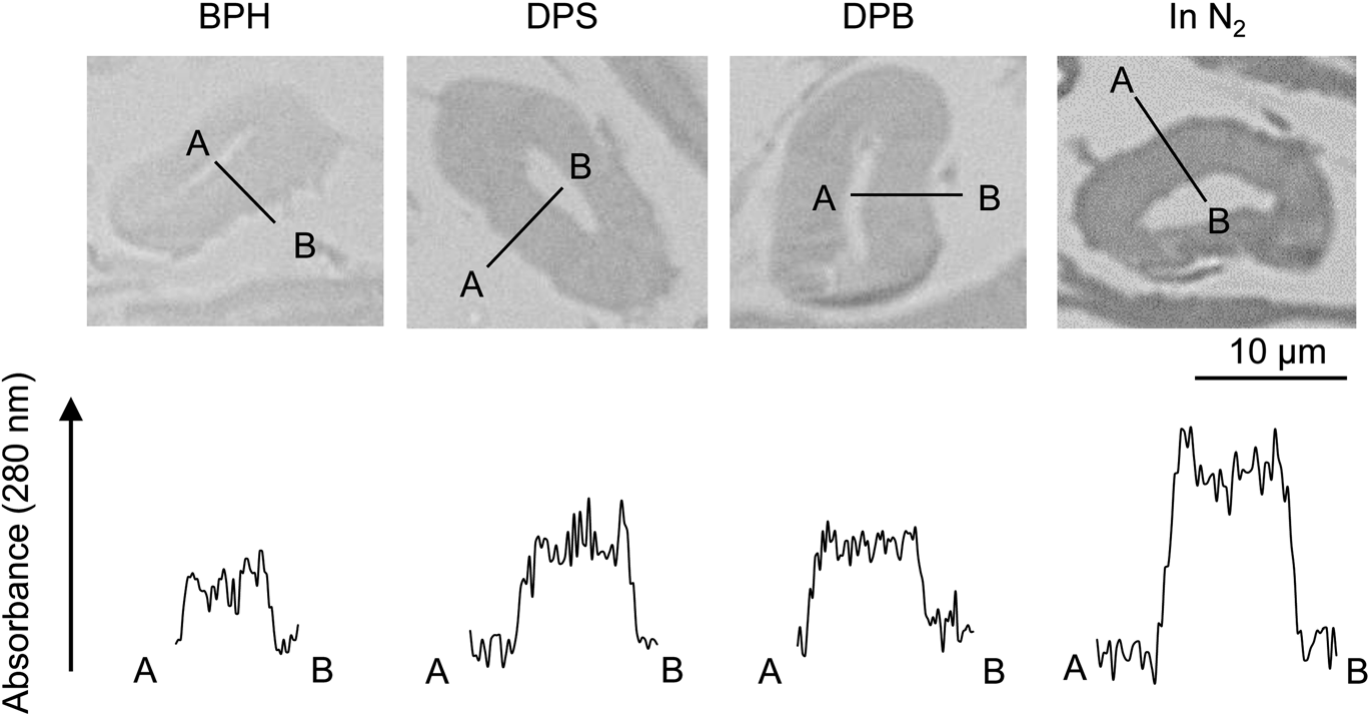
UV absorbance distribution at 280 nm along the cell wall thickness after pyrolysis of cellulose in nitrogen and aromatic solvents at 280 °C.

Fig. 7 compares the UV absorbance at 280 nm in the radial direction of the cell wall. The photographs with a wider field of view are also shown in Fig. S1.† Although the variation of the UV absorbance is not small, the cellulose char prepared under nitrogen tends to exhibit greater absorptivity at the surface than in the interior of the cell wall, suggesting that carbonization occurs more efficiently at the surface under nitrogen. By contrast, the absorbance was more homogeneous when pyrolysis was conducted in aromatic solvents.

**Fig. 8:**
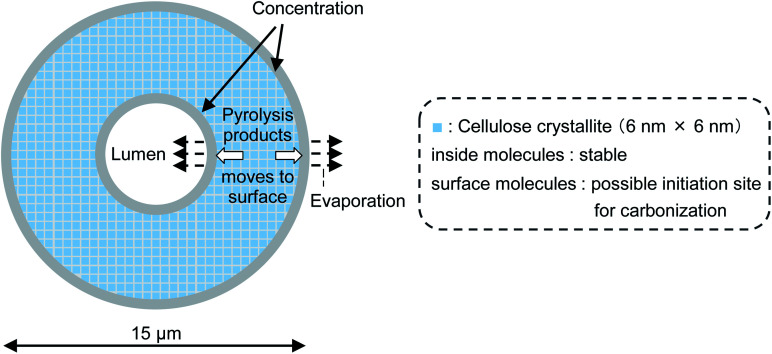
Progress of pyrolysis in cell wall cross section of cotton cellulose fiber in nitrogen to account for accumulation of carbonized products on the surface.

Fig. 8 (not to scale) illustrates the carbonization mechanism of cellulose cell walls. As described previously, the cell walls are hundreds of microcrystals thick. X-ray diffraction analyses of cellulose pyrolysis indicated that cellulose molecules inside the crystallites are stable and that thermal degradation begins with surface molecules.^21,23,37^ It is also known that there is an induction period before weight loss starts during the isothermal heating of cellulose.^38–40^ The formation of “active cellulose” has been proposed as an initiation step to explain the induction period.^38^ Because the induction period was extended by removal of the cellulose reducing end by NaBH_4_ reduction or thermal glycosylation with glycerin, it has been proposed that pyrolysis of the reactive reducing end on the crystal surface initiates the thermal decomposition of cellulose.^40^ Nucleation kinetic models have been proposed for such heterogeneous pyrolysis of cellulose.^41–44^

Liquid levoglucosan (melting point: 183 °C, boiling point: 385 °C ref. 45) is generated at the surfaces of crystallites inside the cell wall, moves to the cell wall surface, and then evaporates at 280 °C. This increases the concentration of levoglucosan at the surface of the cell wall in a manner similar to the higher concentration of pigment that accumulates at the edges when paper soaked in aqueous pigment is dried at ambient temperature. During transport to the surface, levoglucosan undergoes a secondary pyrolysis reaction, producing solid carbonized products inside and at the edges of the cell wall.^36,46^ The behavior of 5-HMF, another intermediate in the formation of solid carbonized product, is similar except that it has higher secondary pyrolysis reactivity than levoglucosan.^16^

**Fig. 9:**
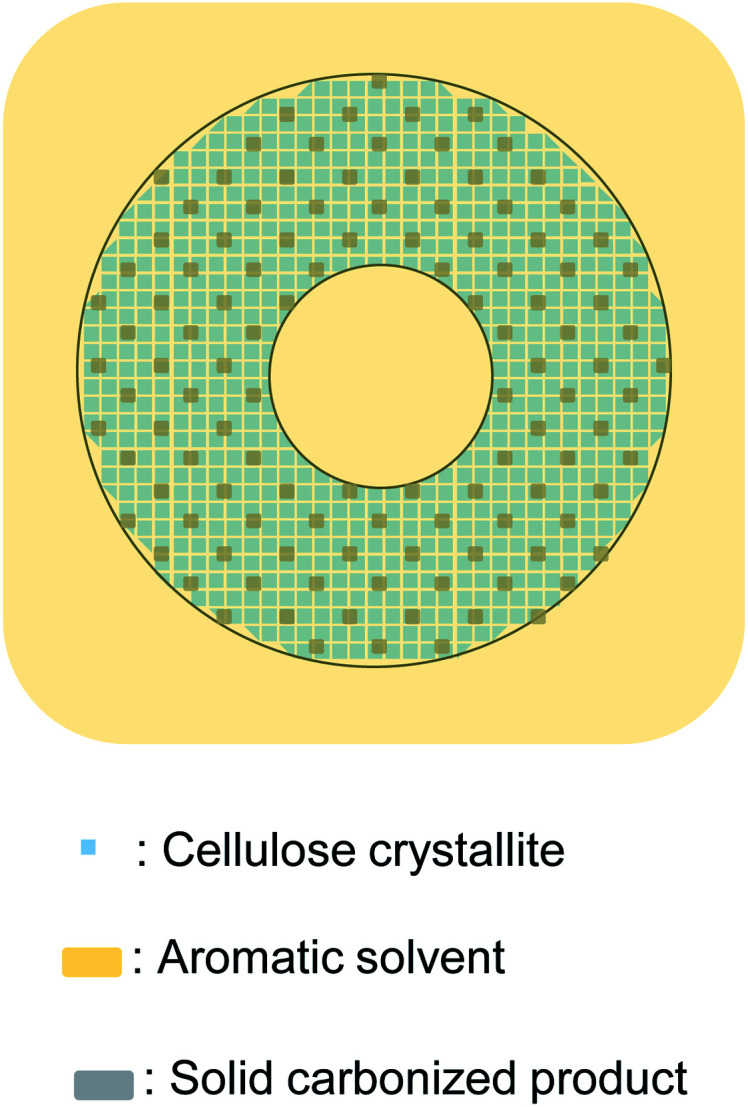
Proposed formation mechanism of carbonized product in cell walls composed of nanocrystallites and the influence of aromatic solvent.

When pyrolyzed in aromatic solvents, solvent penetrates into the cell wall and inhibits the formation of solid carbonized product (Fig. 9). The thermal degradation products dissolve in the solvent and are removed from the cell wall. Because no evaporation is involved, intermediates do not accumulate on the cell wall surface, resulting in homogeneous UV absorbance in the cell wall. Variations in the efficiency of aromatic solvents depend on the ability of the solvent to penetrate the cell wall. Cell wall cellulose molecules that are inaccessible to aromatic solvents decompose in the same way as they do when pyrolyzed under nitrogen. The homogeneity in UV absorbance also suggests that the inaccessible parts of the cell wall are distributed in clusters smaller than the resolution of the UV microscope (280 nm).

The results reported in this work show that controlling the pyrolysis of cellulose and other lignocellulosic biomass requires that cell wall nanostructures derived from cellulose crystallites be taken into account.

## 4. Conclusions

Pyrolysis of cotton cellulose under nitrogen or in aromatic solvents at 280 °C was investigated with UV microscopy. The following conclusions were reached.

(1) Cross sections of cell walls of cellulose char absorb UV because of the presence of furan and benzene rings in the solid carbonized products.

(2) UV absorbance is uniform inside the cell wall except for the surface, where the UV absorbance tends to be stronger. This indicates that the carbonization occurs uniformly inside the cell wall within the resolution of the UV microscope used (280 nm). However, carbonization occurs more strongly at the surface of the cell wall.

(3) A mechanism for the carbonization of cellulose cell walls composed of nano-sized microcrystals has been proposed. Levoglucosan and 5-HMF play important roles as intermediates in cellulose carbonization.

(4) Aromatic solvents uniformly inhibit the formation of solid carbonized product within the cell wall during pyrolysis, probably by inhibiting the secondary pyrolysis reactions of levoglucosan and 5-HMF *via* complex formation. The inaccessible part of the cell wall is carbonized and distributed in the cell wall within the resolution of UV microscope (280 nm).

(5) The characteristic pyrolysis behavior of cellulose cell wall originating from the nature of nano-sized composites of the crystallites is presented. This must be considered to understand and control pyrolysis reactions of cellulose and other lignocellulosic biomass.

## Conflicts of interest

There are no conflicts to declare.

## Supplementary Material

RA-010-C9RA09435K-s001
