# Supplementary material for: Carbonization of cellulose cell wall evaluated with ultraviolet microscopy
Source: RSC Adv. 2020 Feb 19;10(13):7460–7. doi: 10.1039/c9ra09435k (PMC9049861; doi:10.1039/c9ra09435k)
Supplement: RA-010-C9RA09435K-s001 [file RA-010-C9RA09435K-s001.pdf]

Electronic Supplementary Material (ESI) for RSC Advances.
This journal is © The Royal Society of Chemistry 2020

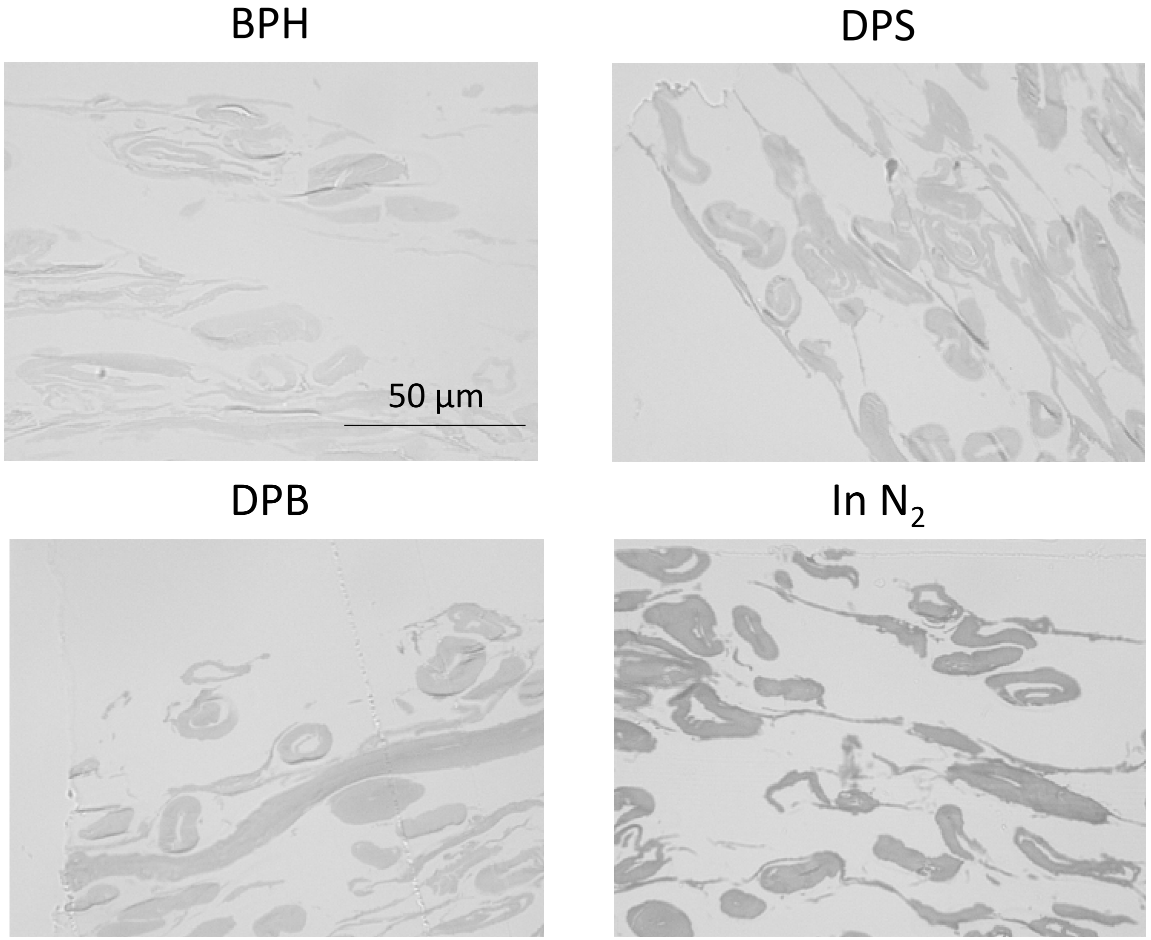

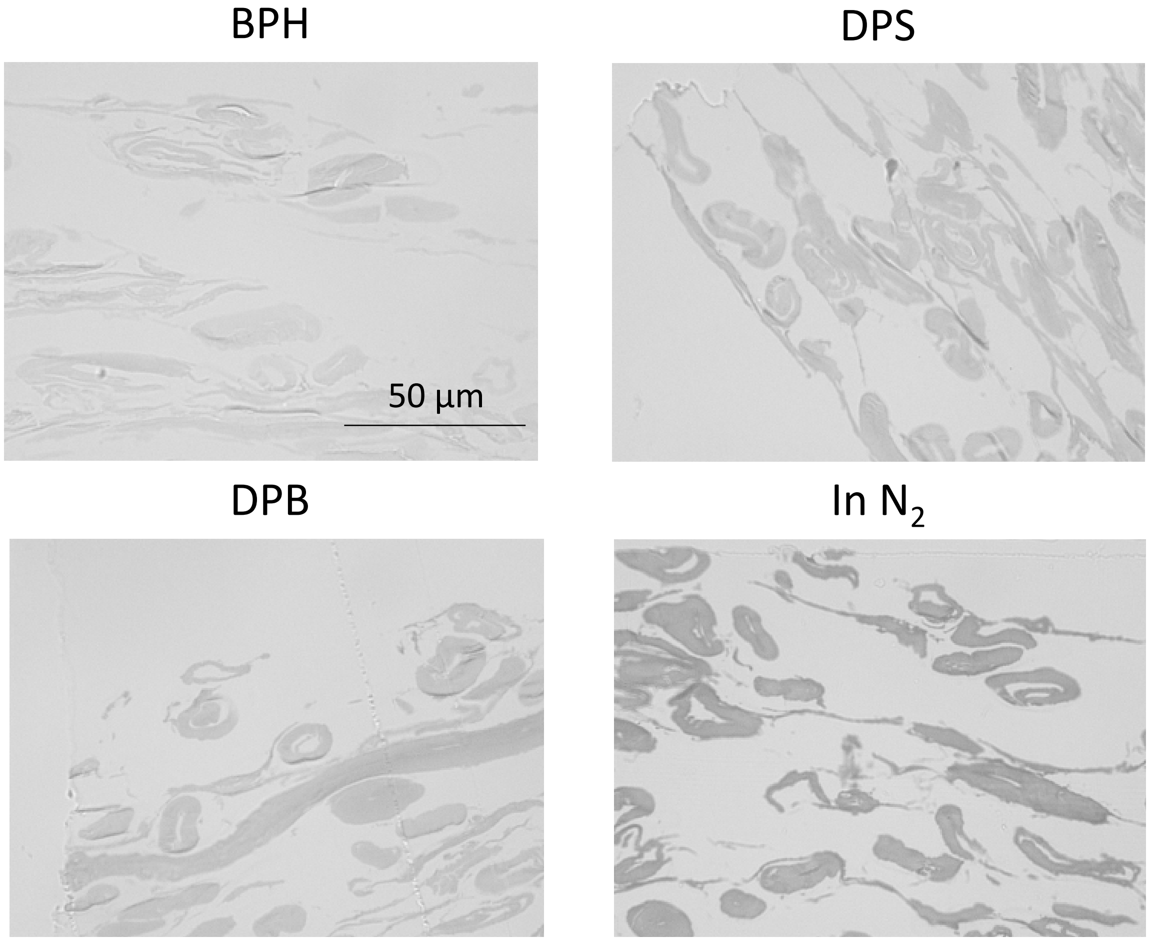

Fig. S1 A wider field of UV microscopic image (280 nm) of pyrolyzed cellulose in nitrogen and aromatic solvents at 280 °C
